# Invasive Community-Acquired Methicillin-Resistant* Staphylococcus aureus* in a Japanese Girl with Disseminating Multiple Organ Infection: A Case Report and Review of Japanese Pediatric Cases

**DOI:** 10.1155/2015/291025

**Published:** 2015-12-27

**Authors:** Ryuta Yonezawa, Tsukasa Kuwana, Kengo Kawamura, Yasuji Inamo

**Affiliations:** ^1^Department of Pediatrics and Child Health, Nihon University School of Medicine, 30-1 Oyaguchi-kamimachi, Itabashi-ku, Tokyo 173-8610, Japan; ^2^Department of Emergency and Critical Care Medicine, Nihon University School of Medicine, 30-1 Oyaguchi-kamimachi, Itabashi-ku, Tokyo 173-8610, Japan

## Abstract

Pediatric invasive community-acquired methicillin-resistant* Staphylococcus aureus* (CA-MRSA) infection is very serious and occasionally fatal. This infectious disease is still a relatively rare and unfamiliar infectious disease in Japan. We report a positive outcome in a 23-month-old Japanese girl with meningitis, osteomyelitis, fasciitis, necrotizing pneumonia, urinary tract infection, and bacteremia due to CA-MRSA treated with linezolid. PCR testing of the CA-MRSA strain was positive for PVL and staphylococcal enterotoxin b and negative for ACME. SCC* mec* was type IVa. This case underscores the selection of effective combinations of antimicrobial agents for its treatment. We need to be aware of invasive CA-MRSA infection, which rapidly progresses with a serious clinical course, because the incidence of the disease may be increasing in Japan.

## 1. Introduction

Community-acquired methicillin-resistant* Staphylococcus aureus* (CA-MRSA) has bacteriological and clinical properties different to those of hospital-acquired MRSA. CA-MRSA is classified into staphylococcal cassette chromosome* mec* (SCC* mec*) type IV or type V, which codes a methicillin resistance. The Panton-Valentine leucocidin (PVL) gene of CA-MRSA sometimes occurs, but there is a low occurrence of positivity to this gene in Japan [1]. This infectious disease is very serious and occasionally fatal, regardless of age. However, invasive CA-MRSA infection is still a relatively rare and unfamiliar infectious disease in Japan.

We report the first successfully positive outcome in a 23-month-old Japanese girl with meningitis, osteomyelitis, fasciitis, necrotizing pneumonia, urinary tract infection, and sepsis due to CA-MRSA.

## 2. Case Presentation

A 23-month-old Japanese girl had presented with a generalized tonic convulsion and pyrexia at a community-based emergency department (ED) 3 days before presentation at our hospital. Her past medical history was unremarkable and she had not recently been hospitalized. On clinical examination at the previous hospital, she had a white cell count of 4900/*μ*L (stab 17.0%, seg 50.0%, lymph 25.0%, and mono 5.0%) and a C-reactive protein level of 5.88 mg/dL (normal level <0.15 mg/dL). Cerebrospinal fluid (CSF) analysis had been performed with no abnormalities. She was treated with cefotaxime (CTX).

The following morning, MRSA was detected in her blood and urine, and the antimicrobial agent was changed from CTX to vancomycin (VCM) and meropenem (MEPM). However, the CSF was negative. On the third day after admission, she developed swelling in the right femur. Magnetic resonance imaging (MRI) of the right femur revealed necrotizing fasciitis.

**Figure 1:**
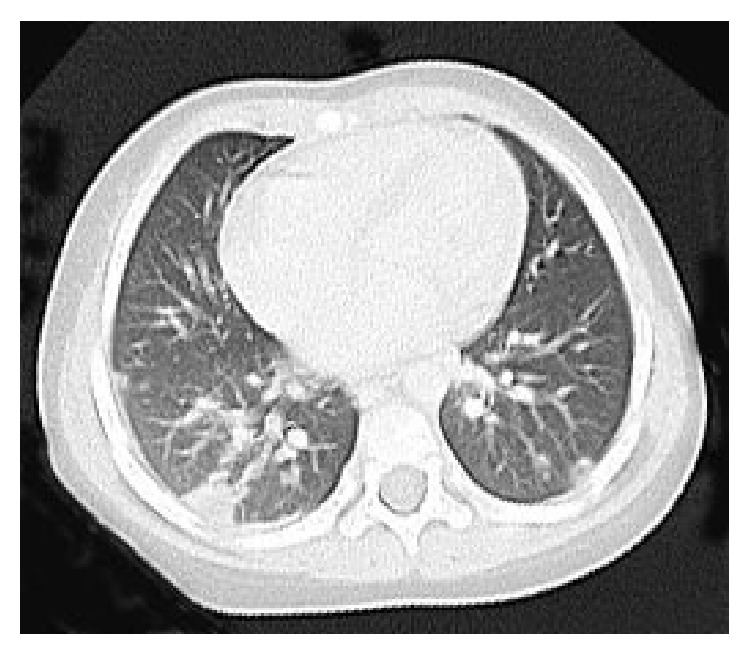
Computed tomography scan of the lungs without contrast showing necrotizing pneumonia with multiple nodules and pleural effusion on admission.

**Figure 2:**
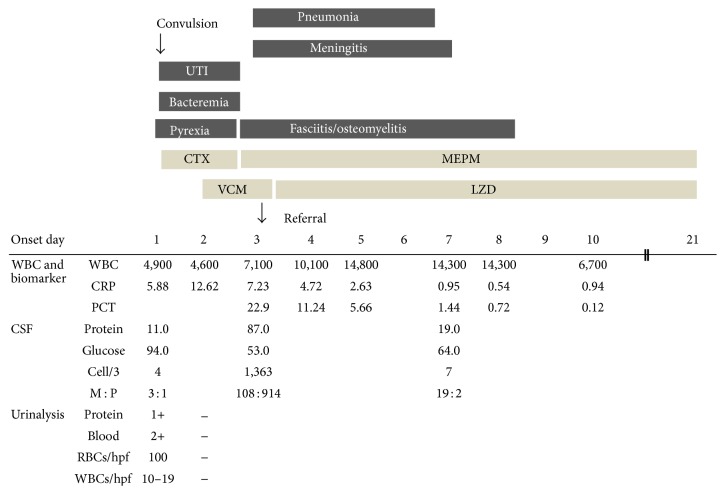
Clinical course of a 23-month-old Japanese girl with meningitis, osteomyelitis, fasciitis, necrotizing pneumonia, urinary tract infection, and sepsis due to community-acquired methicillin-resistant* Staphylococcus aureus*. UTI, urinary tract infection; PCT, procalcitonin; VCM, vancomycin (55 mg/kg/day); LZD, linezolid (30 mg/kg/day); CTX, cefotaxime (100 mg/kg/day); MEPM, meropenem (120 mg/kg/day).

She was transferred for further treatment to our hospital at a tertiary emergency department. A computed tomography (CT) scan of the lungs without contrast on admission showed necrotizing pneumonia with multiple nodules and pleural effusion (Figure 1). A CT scan of the brain without contrast was normal. A transthoracic echocardiography did not reveal vegetation or pulmonary embolisms. CSF analysis was performed again and showed a white cell count of 13,603 cells with 914 polymorphonuclear cells/mL, a total protein level of 0.87 g/dL, and a glucose concentration of 50.3 mg/mL (Figure 2). The CSF culture grew MRSA.

**Figure 3:**
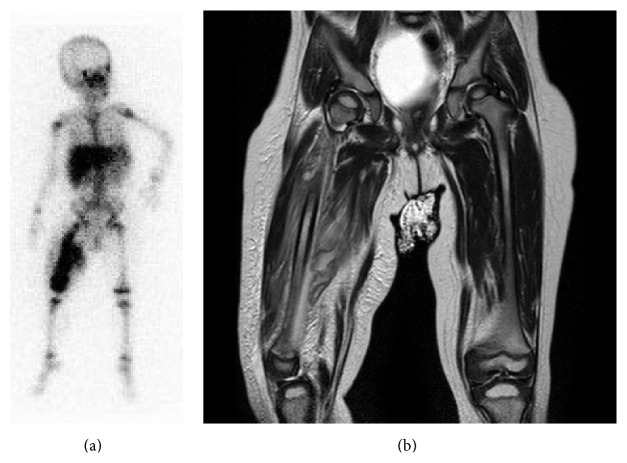
Gallium-67 citrate scintigraphy showing isolated uptake of the right femur (a) and magnetic resonance imaging scans of the right femur showing osteomyelitis with necrotizing fasciitis (T2-weighted image). There are signal changes in the diaphyseal region of the femurs with circumferential soft tissue involvement (b).

LZD and MEPM were started at a dose of 10 mg/kg/day intravenously (IV) every 8 h and 40 mg/kg IV three times daily, respectively. After 3 days of gradual clinical improvement, a gallium-67 citrate scintigraphy and MRI were performed and both showed osteomyelitis of the right femur with necrotizing fasciitis (Figure 3). We continued both antimicrobials until the various infectious lesions had recovered. After 3 weeks of antibiotic therapy, the patient was doing well with significant clinical improvement and no relapse of the infection at 3 months' follow-up. She had no sequelae of invasive CA-MRSA infection or adverse events from the antimicrobials.

*Antimicrobial Susceptibility and Genotype Characterization of MRSA Strain.* CSF culture grew MRSA. The strain was sensitive to arbekacin, gentamicin, VCM, linezolid (LZD), daptomycin, sulfamethoxazole/trimethoprim, and levofloxacin and resistant to oxacillin, using the broth microdilution method. VCM, LZD, and MEPM MICs were 1.0, 1.0, and 0.25 *μ*g/mL, respectively. Polymerase chain reaction testing of the MRSA strain was positive for Panton-Valentine leucocidin (PVL) and staphylococcal enterotoxin b (*seb*) and negative for arginine catabolic mobile element (ACME). The staphylococcal cassette chromosome (SCC)* mec* type was IVa.

## 3. Discussion

CA-MRSA has bacteriological and clinical properties different to those of hospital-acquired MRSA (HA-MRSA). HA-MRSA infections are usually defined as MRSA infection in a patient with one of the following risk factors for HA-MRSA: isolation of MRSA ≥2 days after hospitalization; a history of hospitalization, surgery, dialysis, or residence in a long-term care facility within 1 year before the MRSA-culture date; the presence of a permanent indwelling catheter or percutaneous medical procedure at the time of culture; or previous isolation of MRSA [2]. HA-MRSA strains tend to have multidrug resistance and carry SCC* mec* type II or SCC* mec* type III [3]. HA-MRSA strains are typified by a USA100 or USA200 pulsed field gel electrophoresis pattern [4].

CA-MRSA commonly causes skin and soft tissue infections in previously healthy children. CA-MRSA infection is well known in many countries and has become common in the United States, Taiwan, Canada, European countries, and Australia. CA-MRSA is also classified into SCC* mec* type IV or type V, which codes a methicillin resistance. A Japanese nationwide survey showed the incidence of SCC* mec* type IV in positive CA-MRSA as only 2.3% [1]. Japanese ST8 CA-MRSA with SCC* mec* type IVl (ST8 CA-MRSA/J) has emerged in Japan since 2003 and become well adapted to the Japanese community. This strain was negative for PVL and ACME and positive for superantigen (*spa* variants, SaPI), but not enterotoxin gene cluster, egc (*seg*,* sei*,* sem*,* sen*, and* seo*) and enterotoxin u (*seu*). Most strains were resistant to gentamicin [5]. CA-MRSA clones have been genetically reported among countries: ST1-IV (USA400), ST8-IV (USA300), ST30-IV (Southwest Pacific clone also predominant in Japan), ST59 (Taiwan clone), and ST80 (European clone) clones have been predominant.

There are reports of pediatric deaths from Waterhouse-Friderichsen syndrome [6] and pediatric death from CA-MRSA-associated meningitis in the literature [7].

It is important to prevent more severe and disseminated multiple organ involvement because about 20% of invasive CA-MRSA infection remains bacteremic [8].

Despite the PVL, which is known as a strong virulence factor, invasive CA-MRSA infectious disease is severe regardless of PVL positivity or negativity [9, 10] and very serious and occasionally fatal, regardless of age. However, invasive CA-MRSA infection is still a relatively rare and unfamiliar infectious disease in Japan and even in countries that are experienced in CA-MRSA infection [11, 12].

Invasive CA-MRSA infection should be suspected when CA-MRSA is detected from sterile samples such as blood, CSF, and pleural effusion. The disease, which leads to complications such as meningitis, osteomyelitis, fasciitis, necrotizing pneumonia, urinary tract infection, and sepsis, is a critical and serious infection.

Although invasive CA-MRSA infection is still rare worldwide, the infectious disease incidence has gradually increased. The occurrence of pediatric invasive CA-MRSA infectious disease in the United States was 1.1/100,000 persons/year in 2005 and 1.7/100,000 persons/year in 2010 [8]. However, pediatric invasive CA-MRSA infectious disease is rare in Japan and it is difficult to measure its correspondence to the incidence of CA-MRSA infection. The first pediatric case report of PVL-positive CA-MRSA in Japan was of the death of a 1-year-old patient with severe pneumonia in 2008 [13], the second involved a case report of the death of a 2-year-old patient with severe pneumonia due to PVL-positive CA-MRSA [14], and the third case was the current case involving a positive outcome (Table 1).

The MRSA strain isolated in our study, which was positive for PVL and staphylococcal enterotoxin b (*seb*) and negative for ACME, is a rare molecular characteristic and had not been described before in Japan.

Interestingly, the MRSA strain of the first Japanese case report was positive for PVL and staphylococcal enterotoxin u (*seu*) and enterotoxin gene cluster including seg, sei, sem, sen, and seo genes and negative for ACME [13]. We emphasize that neither our case nor the first case was ST8 CA-MRSA/J. It is possible that this particular isolate has a greater propensity for developing invasive CA-MRSA infection in that it secretes double virulence factors, which are PVL and superantigen.

When infection affects multiple organs in a clinical course, invasive CA-MRSA infection should be considered. Early detection of invasive CA-MRSA in clinical practice is important to prevent a progressively worse outcome. Necrotizing pneumonia, which is caused by invasive CA-MRSA, rapidly develops to respiratory failure with multiple cavities and nodules and pleural effusion in chest X-ray imaging. Invasive CA-MRSA infection is characterized by frequent complications of osteomyelitis and soft tissue infection. When MRSA is detected, we can determine whether it is CA-MRSA according to the circumstances of MRSA detection, for example, from outpatients, detection within 48 hours of admission, no history of MRSA carrier, little antimicrobial response of skin/soft tissue infection, or outbreaks of MRSA in families, nurseries, or sports clubs. The antimicrobial sensitivity of the detected MRSA is different from HA-MRSA and should be rediagnosed as CA-MRSA.

Antibiotics are important to prevent more severe and disseminated multiple organ involvement. Another purpose of antimicrobial therapy is to inhibit production of enterotoxins and PVL, so initial selection of antibiotics can influence the outcome. However, in general, invasive CA-MRSA infection is not familiar to ED physicians. Although early suspicion and/or detection of a rapidly progressive invasive CA-MRSA in a previously healthy child in emergency practice is important, initial empirical therapy for common pediatric infectious diseases in Japan is penicillin or cephalosporin. If a patient suffers from more than one organ infection such as necrotizing pneumonia and fasciitis at their first visit, invasive CA-MRSA is strongly suspected. Empiric therapy such as anti-MRSA antibiotics may be considered [15].

The more severe invasive infections complicated with meningitis have been successfully treated with LZD but not VCM in some case reports [16–20]. Our patient's CSF analysis and CSF culture had no abnormalities at the time of illness onset despite the presence of convulsions. However, meningitis was detected after 3 days during VCM therapy. Meningitis may have already been developing at the time of the convulsions and was not prevented by VCM. Although there is little consensus for administering LZD [15] and it is used in an off-label manner for meningitis and osteoarticular infections in Japan, ED physicians should consider LZD on VCM failure. VCM is common for meningitis, but its CSF penetration (CSF to serum ratios) is not particularly high. The percentage of penetration of CSF to serum is 20% in the nonmeningitis phase and 50% in the active meningitis phase [16]. The mainstay of treatment traditionally has been VCM, but there have been a few cases of VCM failure. Data on patients with meningitis treated with LZD therapy are limited, but the LZD percentage penetration of CSF to serum is 70%. There are some cases of complete recovery from meningitis with LZD treatment [21]. Although VCM is the first choice for invasive CA-MRSA infection, LZD may be a better first-line choice for meningitis or in the presence of CNS signs including convulsion and unconsciousness and symptoms of meningitis prior to development of the disease from a viewpoint of CSF penetration.

Although VCM is a first-line antibiotic in pediatric CA-MRSA infection and LZD is second line, there is still no consensus on the management of severe invasive infection. Because there are no published data to generalize recommendation of linezolid use in pediatric meningitis, a clinical study of LZD in CA-MRSA meningitis should be performed to collect prospective data on CA-MRSA meningitis patients and to further determine the efficacy of LZD treatment of CA-MRSA meningitis.

In conclusion, we reported the diagnosis, management, and successful outcome of a female patient with rapidly progressive and severe invasive CA-MRSA infection. The ED physician should be aware of invasive CA-MRSA infection, which follows a life-threatening clinical course, because appropriate antimicrobial treatment is crucial for improving the prognosis.

## Figures and Tables

**Table 1:** The review of Japanese pediatric cases with severe CA-MRSA infection.

	Case 1	Case 2	Our case
Age	16-month-old boy	24-month-old boy	23-month-old girl

Reported year	2006	2013	2014

Meningitis	−	−	+

Necrotizing pneumonia	+	+	+

Urinary tract infection	−	−	+

Osteomyelitis	−	−	+

Fasciitis	−	−	+

Bacteremia	+	NA	+

Septic shock	+	+	−

Panton-Valentine leucocidin (PVL)	+	+	+

Cassette chromosome *mec* (SCC *mec*)	IVa	NA	IVa

Staphylococcal enterotoxins	*egc* ^*∗*^ (+) *seu* (+)	NA	*seb* (+)

Arginine catabolic mobile element (ACME)	Negative	NA	Negative

Initial antimicrobials	SBT/ABPC + CTX	ABPC	CTX

Antimicrobials after MRSA determined	VCM + MEPM	Already dead	VCM/LZD + MEPM

Outcome	Died	Died	Survived

*egc* ^*∗*^, enterotoxin gene cluster, including seg, sei, sem, sen, and seo genes.

SBT/ABPC, sulbactam/ampicillin; CTX, cefotaxime; MEPM, meropenem; LZD, linezolid; VCM, vancomycin.

## Abbreviations

MRSA: Methicillin-resistant* Staphylococcus aureus*
CA-MRSA: Community-acquired methicillin-resistant* S. aureus*
CTX: Cefotaxime
PVL: Panton-Valentine leucocidin
ACME: Arginine catabolic mobile element
VCM: Vancomycin
MEPM: Meropenem
LZD: Linezolid.
